# Fecal Pathogenic Bacteria Composition and Community Assembly Mechanisms in Captive Brown Bears and Asiatic Black Bears

**DOI:** 10.3390/ani16182875

**Published:** 2026-09-12

**Authors:** Xiaoyang Wu, Xibao Wang, Yongquan Shang, Yao Chen, Ying Li, Ying Gao, Guanglin Zhi, Weilai Sha, Honghai Zhang

**Affiliations:** 1 Shangdong Key Laboratory of Wetland Ecology and Biodiversity Conservation in the Lower Yellow River, College of Life Sciences, Qufu Normal University, Qufu 273165, China; wuxiaoyang1988@126.com (X.W.); wangxibao1995@163.com (X.W.); chenyao92163@163.com (Y.C.); shaweilai@163.com (W.S.); 2 School of Resources and Environment, College of Carbonneutrality, Linyi University, Linyi 276012, China; yongquanshang@163.com; 3 Jinan Wildlife World, Jinan 250215, China; liying1011@126.com; 4 Baoding Aibao Wildlife World, Baoding 071000, China; gaodeying520@163.com; 5 The Guangzhou Wildlife Research Center, Guangzhou Zoo, Guangzhou 510070, China; zhiguanglin@163.com

**Keywords:** 16S rRNA gene sequencing, captive environment, pathogenic bacteria, zoonotic pathogens

## Abstract

Asian black bears (*Ursus thibetanus*) and brown bears (*Ursus arctos*) are commonly kept in captivity in zoos, where captive conditions may alter fecal pathogenic bacterial communities and affect host health, although systematic studies remain limited. This study used 16S rRNA sequencing to characterize the composition, diversity, and community assembly mechanisms of fecal pathogenic bacteria in captive brown bears and black bears. Brown bear samples clustered relatively closely, whereas black bear samples were more dispersed, with juveniles and adults clearly separated; the Shannon index of adult black bears was significantly higher than that of brown bears (*p* < 0.001). Animal pathogenic bacteria (3.37–80.30%) and zoonotic potentially pathogenic bacterial taxa (18.00–96.60%) were the dominant types in both species, with *Escherichia coli* showing the highest abundance in most samples, and zoonotic potentially pathogenic taxa abundance was higher in adults (83.67%) than in juveniles (74.77%). These findings indicate that captivity and host age shape fecal MBPD-annotated potentially pathogenic bacterial communities in ursids, with enrichment of MBPD-annotated zoonotic potentially pathogenic taxa, including *E. coli*–assigned sequences, providing a scientific basis for health management of captive bears and potential public health risk assessment.

## Simple Summary

Black bears and brown bears are commonly kept in zoos, but the composition of fecal bacteria annotated as potentially pathogenic remains incompletely characterized. This study examined fecal bacterial communities in captive brown bears and Asiatic black bears using 16S rRNA gene sequencing and compared adult and juvenile Asiatic black bears. The community composition of MBPD-annotated potentially pathogenic bacterial taxa showed different distribution patterns among the study groups. *Escherichia coli*-assigned sequences were the most abundant MBPD-annotated potentially pathogenic bacterial taxon in most samples. Adult and juvenile Asiatic black bears also showed descriptively different patterns in these taxa. In addition, 16S rRNA gene sequencing and database annotation do not confirm pathogenic strains, virulence, infection, or disease risk. These findings provide baseline descriptive information on fecal MBPD-annotated potentially pathogenic bacterial taxa in the studied captive bears.

## 1. Introduction

Black bears (*Ursus thibetanus*) and brown bears (*U. arctos*) belong to the genus Ursus, family Ursidae, order Carnivora, class Mammalia. As representative taxa of large carnivores, ursids occupy an important place in numerous zoos and wildlife parks owing to their unique ecological status, high public interest, and considerable value for exhibition and science education. In the wild, black bears and brown bears function as apex predators, playing an important role in maintaining ecosystem balance and species diversity [1]. At present, captive breeding and ex situ conservation represent major approaches for the protection and rescue of black bears and brown bears, and both species have long been widely used for science education, species conservation, and public display. However, compared with natural habitats, captive environments differ substantially in spatial extent, activity patterns, dietary composition, and frequency of human contact. Such artificially imposed captive conditions may increase the likelihood of disease or alter the health status of black bears and brown bears (e.g., leading to subclinical or suboptimal health conditions), and may exert profound effects on their fecal microecosystem through multiple pathways [2].

The fecal microbial community of black bears and brown bears constitutes a complex micro-ecosystem composed of bacteria (probiotics and pathogenic bacteria), fungi, archaea, and viruses [3,4]. A high abundance of beneficial bacteria and a stable microbial community structure help maintain fecal micro-ecological balance and facilitate host degradation of indigestible substances [5,6]. However, an increase in the abundance of pathogenic bacteria not only disrupts the structure of the fecal microbial community but also readily induces diseases such as diarrhea and intestinal inflammation in the host [2,7,8]. Among these, zoonotic pathogenic bacteria warrant particular attention: such bacteria can not only compromise the health of the animals themselves but also carry a potential risk of transmission between humans and animals through contact, fecal contamination, and other routes, thereby posing a potential threat to the public health and safety of zoo staff and visitors. *Escherichia coli*, an opportunistic pathogen widely distributed in the intestines of humans and animals, has certain pathogenic subtypes that have been confirmed to be closely associated with various zoonotic infectious diseases, making it a key target that cannot be overlooked in the monitoring of fecal pathogenic bacteria in captive wildlife. Previous studies have shown that captive environments can induce fecal microbiota dysbiosis in animals, accompanied by a significant increase in the abundance of zoonotic pathogenic bacteria [9,10,11].

Previous studies on the fecal microbiota of wild and captive animals have mostly focused on microbial diversity, the compositional structure of dominant phyla/genera, and their associations with host physiological status and dietary structure [12], whereas systematic research on the specific types, abundance distribution, and community assembly mechanisms of fecal pathogenic bacteria remains relatively limited, particularly in captive ursids. Moreover, host age plays an important role in the development and stabilization of the fecal microbiota: the fecal microbiota of juvenile individuals is typically in the early stages of establishment and development, with a relatively unstable community structure [13], which may render them more susceptible to colonization by exogenous microorganisms, including pathogenic bacteria, than adults; however, studies examining age-related differences in fecal pathogenic bacteria among captive bears remain insufficient. Meanwhile, exploring the assembly mechanisms of fecal pathogenic bacterial communities from a community ecology perspective—namely, the relative contributions of stochastic processes (such as migration, dispersal, and random colonization) and deterministic processes (such as host selection and environmental filtering)—can help elucidate how captive environments shape the compositional patterns of host fecal pathogenic bacteria [14]; however, the application of such approaches in the field of fecal pathogenic bacteria in captive wildlife remains limited. The neutral community model (NCM), as a classical theoretical framework for assessing the contribution of stochastic processes, provides an effective analytical tool for addressing the above questions [15].

Based on the above background, this study used fecal samples collected from captive brown bears and captive Asiatic black bears (including both adult and juvenile individuals) in China. Fecal samples were used as non-invasive proxies for the gastrointestinal microbial communities. Through high-throughput sequencing of the V3–V4 region of the 16S rRNA gene, combined with data from the multiple bacterial pathogen detection (MBPD) database, we systematically characterized the composition, taxonomic distribution, and relative abundance of potential pathogenic bacteria detected in fecal samples. We compared the diversity of fecal MBPD-annotated potentially pathogenic bacterial communities between species and among individuals at different life stages and explored the relative contributions of stochastic and deterministic processes to community assembly using the neutral community model. We hypothesized that the composition and diversity of fecal MBPD-annotated potentially pathogenic bacterial communities, as well as the relative contributions of stochastic and deterministic assembly processes, would differ between the two bear species and between adult and juvenile individuals. This study aimed to reveal the potential effects of host species and host age on fecal MBPD-annotated potentially pathogenic bacterial communities in ursids and to provide a theoretical basis for health management and husbandry of captive brown bears and Asiatic black bears. By integrating pathogen-focused community profiling with community assembly analysis, this study provides novel insights into the potential health risks and ecological mechanisms associated with fecal pathogenic bacteria in captive ursids.

## 2. Materials and Methods

### 2.1. Experimental Materials

Fecal samples from brown bears (*Ursus arctos*) and black bears (*Ursus thibetanus*) used in this study were collected from Yangqi Mountain Zoo in Chaohu City, Anhui Province, China, and Jinan Wildlife Zoo in Shandong Province, China, respectively. The dietary structures of brown bears and black bears at the two breeding facilities were reasonably comparable: brown bears were fed primarily on fresh vegetables, various grains, artificial compound feed, and vitamin–mineral premixes, resulting in a relatively balanced nutritional composition, whereas black bears were fed primarily on apples, carrots, and grains. The vegetable and grain items provided at the two facilities were not completely identical; however, both groups received plant-based foods and grains as major dietary components, and no substantial dietary differences were identified between the two groups. All animals were clinically healthy at sampling, had received no antimicrobial treatment during the preceding 3 months, and had not received other medications. Animals were maintained in captive enclosures and co-housed with conspecifics under routine zoo management. Samples were collected in March. Sample collection strictly followed aseptic procedures: collectors wore sterile disposable gloves and used sterile disposable tubes to collect the inner portion of fresh feces that had not been in direct contact with the external environment or the ground, thereby minimizing contamination from environmental microorganisms and exogenous DNA to the greatest extent possible. Immediately after collection, samples were stored in a portable refrigerator (−20 °C) and maintained under a continuous cold chain throughout transport. Upon arrival at the laboratory, samples were uniformly stored in an ultra-low-temperature freezer (−80 °C) prior to DNA extraction, to ensure the integrity and stability of the microbial genetic material within the samples.

A total of 10 brown bear fecal samples (numbered UA1–UA10) and 10 black bear fecal samples (numbered UT1–UT10) were collected in this study. Each fecal sample was collected from one independent individual, and no repeated samples were included. Among the brown bear samples, 6 were from female individuals (UA1–UA6) and 4 from male individuals (UA7–UA10), all 5 years of age; the relatively homogeneous age and sex composition of these individuals helped to reduce the interference of individual factors on the fecal microbiota analysis results. Among the black bear samples, 7 were from male individuals (UT1–UT7) aged between 4 and 4.5 years, while the remaining 3 samples were from juvenile individuals of unknown sex (UT8–UT10) aged only 0.25 years (approximately 3 months old). At the time of sampling, the juvenile black bears were not fully weaned and were still receiving maternal milk in addition to age-appropriate supplementary foods. Detailed sample information is presented in Table A1.

### 2.2. DNA Extraction

Samples were stored at −80 °C for less than 1 month before DNA extraction. Total DNA was extracted from approximately 180 mg of fecal material using the QIAamp Fast DNA Stool Mini Kit (Qiagen, Hilden, Germany) according to the manufacturer’s instructions, strictly following the recommended lysis, binding, washing, and elution steps to ensure the integrity and reproducibility of nucleic acid extraction. The concentration and purity of the extracted DNA samples were assessed using a NanoDrop 2000 spectrophotometer (Thermo Fisher Scientific, Waltham, MA, USA), with DNA quality evaluated by measuring the OD260/280 and OD260/230 ratios to ensure that the samples met the requirements for subsequent PCR amplification and library construction.

### 2.3. PCR Amplification, Library Construction, and Sequencing

The V3–V4 hypervariable region of the bacterial 16S rRNA gene was targeted for amplification using the universal primers 338F (5′-CCTAYGGGRBGCASCAG-3′) and 806R (5′-GGACTACNNGGGTATCTAAT-3′). This region was selected because it provides reliable bacterial community profiling and facilitates comparison with existing fecal microbiome datasets and reference databases. It was appropriate for characterizing the composition and relative abundance of potential pathogenic bacteria in fecal samples. The PCR reaction system had a total volume of 50 µL, comprising 5 µL of template DNA, 25 µL of KAPA HiFi HotStart Ready Mix (Roche, Menlo Park, CA, USA), 10 µL of double-distilled water, and 5 µL each of the forward and reverse primers. Forward and reverse primers were prepared at 10 µM stock solutions and were added to the PCR reaction at a final concentration of 1 µM each (5 µL of each primer per 50 µL reaction). The amplification program was as follows: initial denaturation at 95 °C for 10 min; followed by 25 cycles, each consisting of denaturation at 94 °C for 30 s, annealing at 55 °C for 30 s, and extension at 72 °C for 30 s; and a final extension at 72 °C for 5 min to ensure complete end-filling of the amplification products. After purification, the PCR products were used to construct sequencing libraries with the TruSeq DNA PCR-free Sample Preparation Kit (Illumina, San Diego, CA, USA) according to the manufacturer’s protocol, a method that effectively avoids the introduction of additional PCR bias during library construction. Libraries were indexed using the Illumina-compatible indexing procedure according to the manufacturer’s protocol and sequenced as 2 × 250 bp paired-end reads on the Illumina HiSeq 2500 (Illumina, San Diego, CA, USA).

### 2.4. Raw Data Quality Control and Pathogenic Bacteria Annotation

The raw sequencing data obtained were first subjected to quality control using Fastp software (V 1.0.1) with default parameters to remove low-quality bases and low-quality reads [16], ensuring the reliability of the sequences used in subsequent analyses. EasyAmplicon software (V 2.0) was then used to remove primer sequences and adapter contamination, and paired-end reads were merged to obtain high-quality effective sequences [17]. Primer trimming, paired-end read merging, ASV inference, and chimera removal were performed according to the EasyAmplicon V2.0 workflow [17]. On this basis, the amplicon sequence variant (ASV) approach was employed to cluster and denoise the merged sequences, allowing finer discrimination of sequence variants corresponding to single-base differences and thus achieving higher resolution than traditional OTU clustering methods. To eliminate the influence of differences in sequencing depth on downstream analyses, all samples were rarefied to the sequencing depth of the sample with the lowest read count, thereby providing a consistent basis for comparison across samples [18].

To further identify potential pathogenic bacterial taxa present in the samples, this study employed the multiple bacterial pathogen detection (MBPD) pipeline [19], aligning the obtained ASV representative sequences against the MBPD reference database (V 2022-08-18). Given the limited species- and strain-level resolution of 16S rRNA V3–V4 amplicon sequencing, taxa annotated using the MBPD database were regarded as potentially pathogenic bacterial taxa. Alignment was performed using the UCLUST algorithm with a confidence threshold set at 0.8, ultimately generating the taxonomic classification of pathogenic bacteria in the samples and a relative abundance table at the species level [19]. Based on these results, the tax_stackplot.R script in EasyAmplicon software (V 2.0) was further used to generate stacked bar plots of pathogenic bacterial taxonomic composition and relative abundance at the species level, providing a visual representation of the compositional characteristics of the pathogenic bacterial community across different samples [18].

### 2.5. Analysis of Microbial Diversity and Differences

The α-diversity of the MBPD-annotated potentially pathogenic bacterial community was analyzed using tools provided by the Tutool platform (https://www.cloudtutu.com/), and the Wilcoxon rank-sum test was applied to compare between-group differences in the Richness index (species richness) and Shannon index (community diversity), which were calculated using only ASVs annotated as potentially pathogenic based on the MBPD database. The Wilcoxon rank sum test was applied to compare index distributions between groups. For β-diversity, principal coordinates analysis (PCoA) and non-metric multidimensional scaling (NMDS) were performed based on the Bray–Curtis distance matrix using the amplicon package in R (version 4.5.2). To statistically assess the effects of the grouping variable on β-diversity, a permutational multivariate analysis of variance (PERMANOVA) was conducted using the adonis2 function in the vegan package in R (version 4.5.2). The Bray–Curtis distance matrix was used as the input, the number of permutations was set to 999, and the grouping variable tested was species (brown bear vs. Asiatic black bear). The exact R^2^ and *p* values were reported in the Results for each ordination/metric.

### 2.6. Community Assembly Processes

The Sloan neutral community model (NCM) was used to explore the relationship between the occurrence frequency of taxa across samples and their mean relative abundance in the corresponding metacommunity [15]. The analysis was conducted separately for the UA, UTA, and UTY groups using the ASV abundance table of MBPD-annotated potentially pathogenic bacterial taxa. For each group, the metacommunity was defined as the pooled MBPD-annotated potentially pathogenic bacterial ASV community across all samples within that group. The input data were organized as an ASV-by-sample abundance matrix. No additional abundance- or prevalence-based filtering was applied before the analysis. For each ASV, occurrence frequency was represented by the proportion of samples in which it was detected, whereas mean relative abundance was calculated across samples within the corresponding group.

The analysis was performed using the NCM module of the Tutu online analysis platform (https://www.cloudtutu.com/). The output displayed the relationship between occurrence frequency and mean relative abundance, together with the fitted neutral expectation and 95% prediction boundaries. ASVs located above, within, or below the prediction boundaries were descriptively interpreted as occurring more frequently than predicted, consistent with the fitted neutral expectation, or less frequently than predicted, respectively. The platform output also reported the composite parameter Nm, where N represents the local community size and m represents the immigration probability, and an R^2^ value representing model fit. The devtools and ggimage packages in R (version 4.5.2) were used for package management and graphical presentation, respectively.

## 3. Results

### 3.1. Overview of Sequencing Data

High-throughput sequencing of the 16S rRNA gene V3–V4 region was performed on a total of 20 fecal samples from Asiatic black bears (group UT) and brown bears (group UA). After quality control, a total of 1,229,835 effective sequences were obtained, with an average of 61,491.75 effective sequences per sample. The overall sequence yield was sufficient, providing a solid data foundation for the subsequent analysis of fecal pathogenic bacterial community structure. Following ASV denoising and chimera removal, a total of 823 ASVs were clustered across all samples, reflecting a relatively rich diversity of pathogenic bacterial taxa in the feces of captive Asiatic black bears and brown bears. At the individual sample level, the number of ASVs varied to some extent among samples: the black bear sample UT5 harbored the highest number of ASVs (341), whereas the brown bear sample UA4 harbored the lowest (63). This suggests potential heterogeneity in the composition of fecal MBPD-annotated potentially pathogenic bacterial communities among different individuals and species, which also provides preliminary clues for the subsequent comparative analysis of diversity between groups.

**Figure 1:**
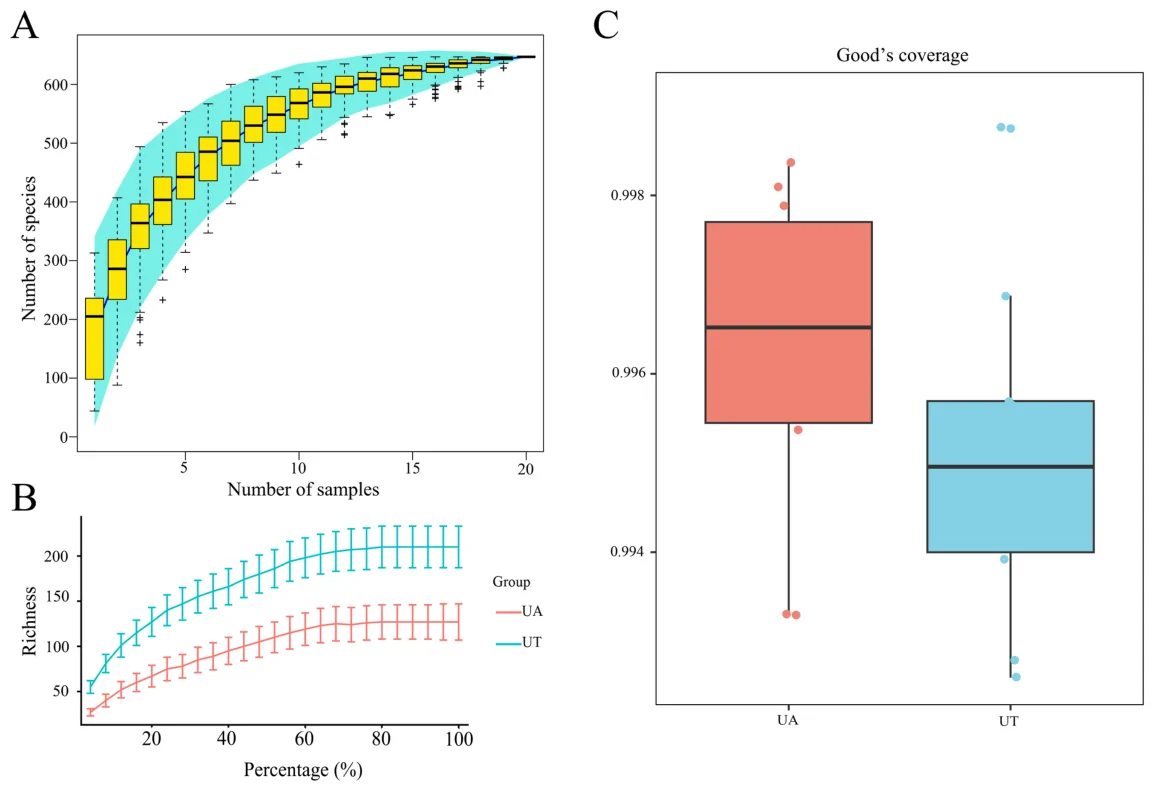
Species accumulation boxplot (**A**), rarefaction curves (**B**), and boxplot of Good’s coverage (**C**).

To assess whether the sequencing depth was sufficient to fully cover the species diversity of the samples, species accumulation box plots (Figure 1A) and rarefaction curves (Figure 1B) were further generated. The results showed that both curves exhibited a pattern of rapid initial increase followed by a gradual plateau as the number of sampled individuals or sequencing depth increased, ultimately reaching a saturation phase. This indicates that the current sequencing depth was sufficient to adequately capture the species richness information of the samples, and that further increases in sequencing volume were not required to meet the data saturation requirements for subsequent statistical analyses.

In addition, the Good’s coverage index for all samples exceeded 99% (Figure 1C), indicating that the sequencing depth was sufficient to reflect, with high confidence, the true microbial species composition present in the samples. The sequencing results are therefore highly representative and reliable, accurately reflecting the actual composition of the fecal MBPD-annotated potentially pathogenic bacterial communities in captive Asiatic black bears (UT) and brown bears (UA), and providing a solid and reliable data foundation for the subsequent comparative analysis of diversity and community structure between the two groups.

### 3.2. Diversity of Fecal Pathogenic Bacteria in Captive Asiatic Black Bears and Brown Bears

**Figure 2:**
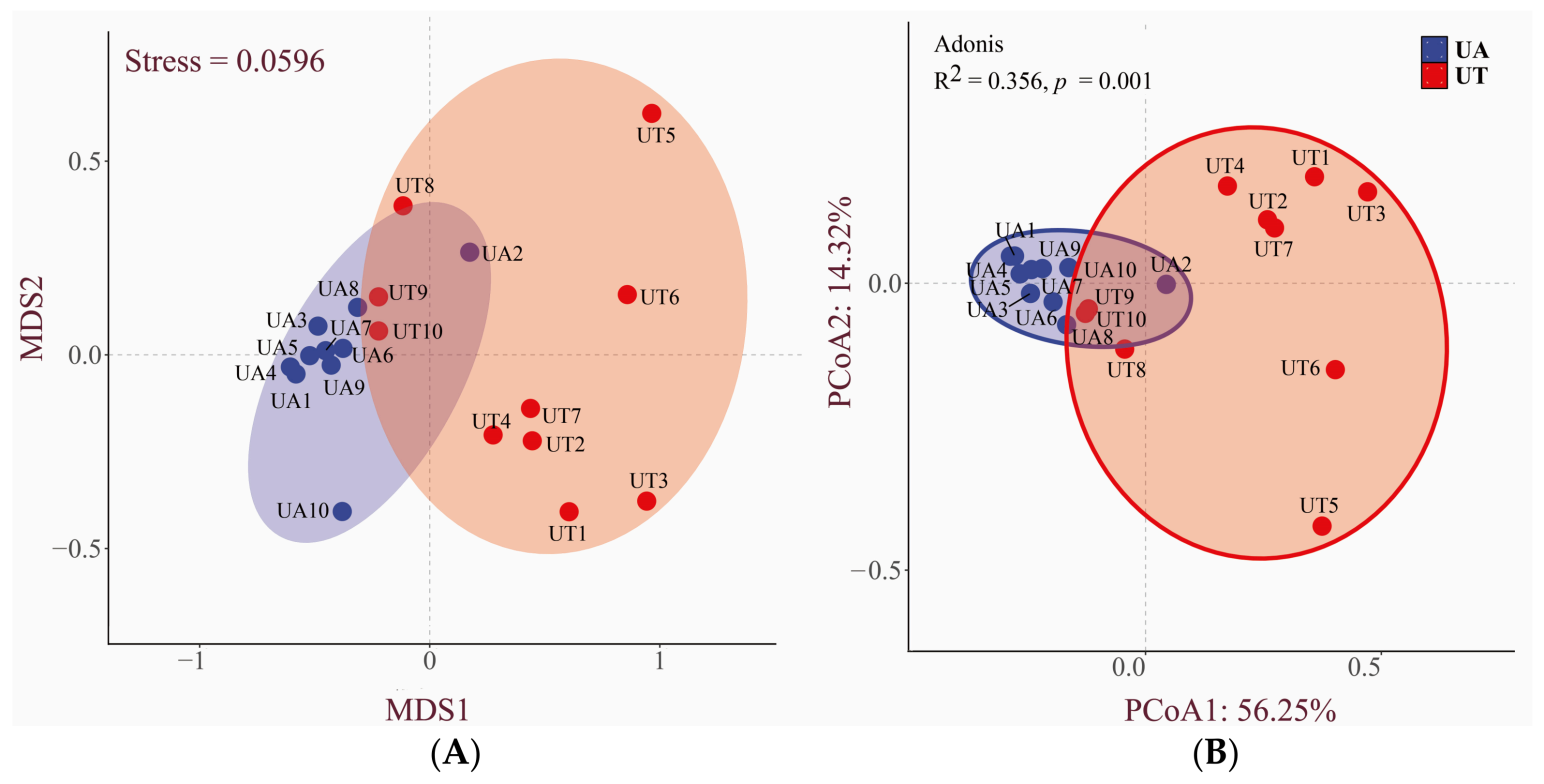
Non-metric multidimensional scaling (**A**), where stress < 0.1 indicates a good ranking, and principal co-ordinates analyses (**B**).

To visualize beta-diversity patterns of the MBPD-annotated potentially MBPD-annotated potentially pathogenic bacterial communities, NMDS (Figure 2A) and PCoA (Figure 2B) were performed based on the Bray–Curtis distance matrix. Moreover, PERMANOVA based on the Bray–Curtis distance matrix indicated that species (brown bear vs. Asiatic black bear) significantly explained the variation in β-diversity (R^2^ = 0.356, *p* = 0.001). The results showed that the sample distribution patterns revealed by the two ordination methods were highly consistent: samples from the brown bear (UA) group were relatively concentrated and tightly clustered in the ordination space, with small inter-sample differences, whereas samples from the Asiatic black bear (UT) group exhibited a markedly more dispersed distribution, indicating pronounced within-group heterogeneity. Notably, the black bear group displayed a clear age-related differentiation pattern: the juvenile black bear samples (UT8, UT9, and UT10) clustered near the origin of the ordination plot and appeared separated in the ordination space from the adult black bear samples (UT1–UT7), the latter showing a much broader distribution along the ordination axes.

**Figure 3:**
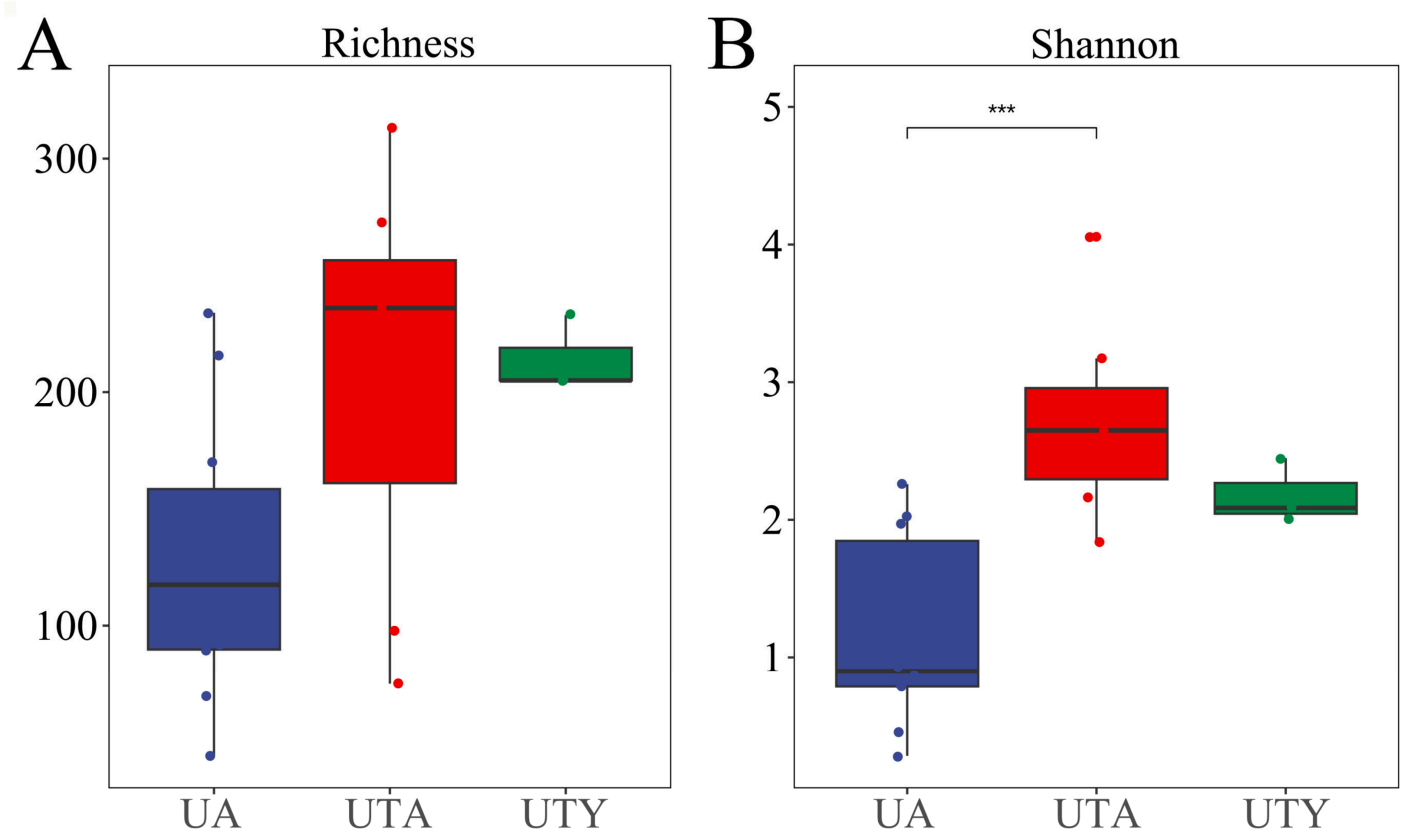
Analysis of intestinal pathogen diversity among three groups ((**A**), Richness index; (**B**), Shannon index; *** *p* < 0.001).

In light of the age-related differentiation pattern revealed by the above ordination analyses, the original black bear group was further subdivided, based on age information, into a juvenile black bear group (UTY, i.e., UT8–UT10) and an adult black bear group (UTA, i.e., UT1–UT7). On this basis, a comparative analysis of α-diversity among the three groups—UTY, UTA, and the brown bear group (UA)—was conducted (Figure 3). For the Richness index (Figure 3A), the UTA group exhibited relatively higher median values and overall distribution compared with the UA and UTY groups, which were both relatively lower. However, according to the Wilcoxon rank-sum test, no statistically significant differences in the Richness index were detected among the captive brown bear, juvenile black bear, and adult black bear groups (*p* > 0.05), suggesting that the species richness of fecal MBPD-annotated potentially pathogenic bacterial communities was generally similar among the three groups and had not yet diverged significantly with age or species. For the Shannon diversity index (Figure 3B), the comparison among the three groups yielded a different pattern from that of the Richness index: the fecal pathogenic bacterial community diversity of the adult black bear (UTA) group was significantly higher than that of the brown bear (UA) group, with an extremely high level of statistical significance (*p* < 0.001); the Shannon index of the juvenile black bear (UTY) group was numerically intermediate between the UA and UTA groups, and although slightly higher than that of the brown bear group, this difference did not reach statistical significance (*p* > 0.05). These results indicate that the significant increase in fecal pathogenic bacterial diversity observed in captive black bears was mainly attributable to adult individuals, whereas the pathogenic bacterial diversity characteristics of juvenile black bears were more similar to those of brown bears and had not yet exhibited the marked elevation observed in adults. This finding also echoes, from the perspective of diversity, the spatial distribution pattern observed in the ordination analyses, in which juvenile black bears and brown bears were shown to be more similar in community structure.

### 3.3. Composition of Fecal Pathogenic Bacteria

**Figure 4:**
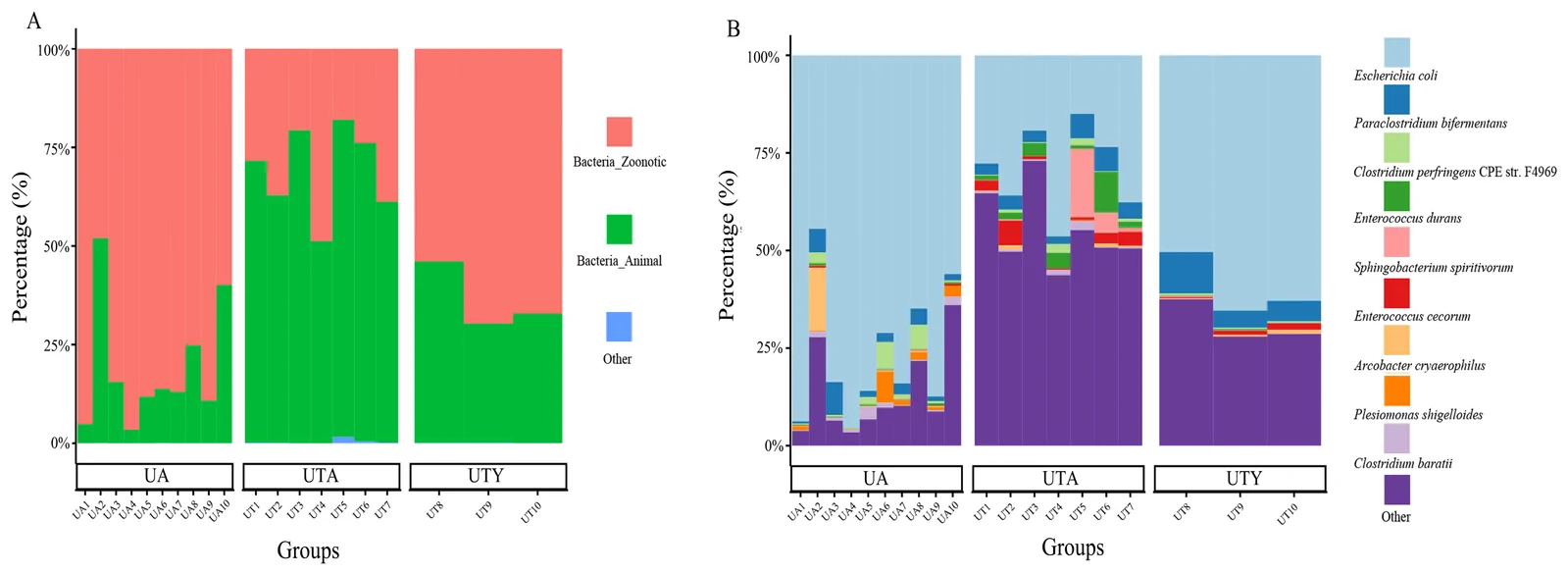
Relative abundance plots of fecal MBPD-annotated potentially pathogenic bacteria at the pathogenic type level (**A**) and species level (**B**) in captive brown bears and black bears.

To further clarify the specific types of pathogenic bacteria present in the feces of captive Asiatic black bears and brown bears and their relative abundance distribution, a taxonomic analysis of the pathogenic bacterial community composition was conducted for each sample (Figure 4). The results showed that the pathogenic bacteria detected in the fecal samples of black bears and brown bears collected from the two zoos could be broadly classified into two major categories: animal pathogenic bacteria (relative abundance ranging from 3.37% to 80.30%) and zoonotic pathogenic bacteria (relative abundance ranging from 18.00% to 96.60%), which together constituted the main components of the pathogenic bacterial community in each sample (Figure 4A).

With respect to differences between species, the mean relative abundance of animal pathogenic bacteria in the feces of captive brown bears (59.07% ± 17.49%) was higher than that of zoonotic pathogenic bacteria (40.65% ± 17.27%), suggesting that the fecal pathogenic bacterial community of brown bears was dominated by animal pathogenic bacteria. In contrast, a clear age-related difference was observed within the captive black bear group: the mean relative abundance of MBPD-annotated zoonotic potentially pathogenic bacterial taxa in the feces of adult black bears (83.67% ± 15.12%) was numerically higher than that in juvenile black bears (74.77% ± 11.97%), suggesting that the proportion of zoonotic pathogenic bacteria in the feces of black bears tends to increase further with age.

At the species taxonomic level, Figure 4B further reveals the specific composition of pathogenic bacterial taxa in each sample. The results showed that, with the exception of the black bear sample UT5, the pathogenic bacterium with the highest relative abundance in all other samples was *Escherichia coli* (a zoonotic pathogenic bacterium), whose relative abundance varied considerably among samples, ranging from 19.30% to 95.50%. This indicates that *E. coli*-assigned sequences were abundant in most samples and represented the most common MBPD-annotated potentially pathogenic taxon detected in the feces of both captive bear populations. However, owing to the limited resolution of 16S rRNA V3–V4 sequencing, this annotation does not confirm the presence of a specific pathogenic E. coli lineage or its virulence potential. A notable exception was observed in sample UT5, in which the pathogenic bacterium with the highest relative abundance was not *E. coli* but *Sphingobacterium spiritivorum* (an animal pathogenic bacterium), with a relative abundance of 17.60%; *E. coli* ranked second in this sample, with a relative abundance of 15.00%. This distinctive pathogenic bacterial composition pattern observed in sample UT5 corroborates its notable deviation from the other black bear samples in the NMDS and PCoA ordination analyses, in which it was distributed independently at the margin of the ordination space. This suggests that the fecal pathogenic bacterial community structure of this individual may exhibit certain distinctive features that warrant further attention in future research.

### 3.4. Assembly Mechanisms of MBPD-Annotated Potentially Pathogenic Bacterial Communities

**Figure 5:**
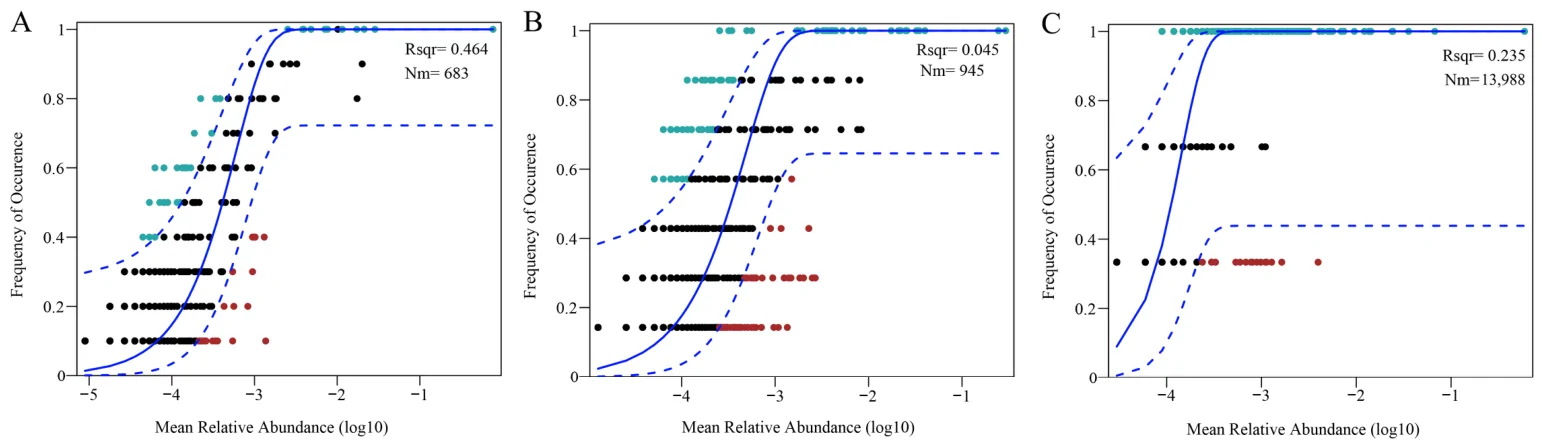
Assembly mechanism of fecal MBPD-annotated potentially pathogenic bacteria in captive brown bears (**A**), adult captive black bears (**B**), and young black bears (**C**).

To exploratorily visualize occurrence–abundance relationships of MBPD-annotated potentially pathogenic bacterial ASVs, the Sloan neutral community model (NCM) was fitted separately for the three groups (UA, UTA, and UTY) (Figure 5). The platform-reported model-fitting values (R^2^) were below 0.5 in all three groups. The R^2^ values were 0.464 for the captive brown bear group (Figure 5A), 0.045 for the captive adult black bear group (Figure 5B), and 0.235 for the captive juvenile black bear group (Figure 5C). The platform-reported composite parameter Nm was 683 for the captive brown bear group (Figure 5A), 945 for the captive adult black bear group (Figure 5B), and 13,988 for the captive juvenile black bear group (Figure 5C). The Nm value was numerically higher in the juvenile black bear group than in the other two groups.

## 4. Discussion

Based on 16S rRNA gene high-throughput sequencing combined with the MBPD pathogenic bacteria database, this study systematically characterized the composition of fecal MBPD-annotated potentially pathogenic bacterial taxa and the occurrence–abundance relationships of these communities in captive brown bears, captive adult black bears, and captive juvenile black bears, providing fundamental data for a deeper understanding of the distribution patterns of fecal MBPD-annotated potentially pathogenic bacterial taxa in bear species under captive conditions.

With respect to MBPD-annotated potentially pathogenic bacterial composition, the relative abundance of zoonotic pathogenic bacteria exceeded 18% in all samples in this study, reaching as high as 96.60% in some samples. Because the annotation was based on 16S rRNA gene sequencing and the MBPD database, these values represent the relative abundance of annotated taxa and do not confirm the presence of pathogenic strains, virulence, infection, or disease. In comparison, previous studies have reported the occurrence of potential pathogenic bacteria in the feces of captive cheetahs [20]. Although host species, diet, geographic location, husbandry conditions, and analytical procedures differ among studies, these findings collectively indicate that potentially pathogenic bacterial taxa can be detected in the fecal microbiota of diverse captive mammals. Although this study was unable to collect fecal samples from wild black bears and brown bears as direct controls, making a strict within-species comparison between wild and captive individuals difficult, the present results characterize the composition and relative abundance of MBPD-annotated potentially pathogenic bacterial taxa in the studied captive brown bear and black bear populations. At the species taxonomic level, *E. coli* exhibited the highest relative abundance among pathogenic bacterial taxa in the vast majority of brown bear and black bear samples, consistent with previous findings of the widespread predominance of *E. coli* in the fecal microbiota of other captive animals [21]. As a typical zoonotic pathogen, *E. coli* is widely present in environmental media such as keepers, visitors, and captive facilities, and its high detection frequency and high abundance in the feces of captive bears further support the hypothesis of a potential association between captive environment exposure and the enrichment of fecal pathogenic bacteria.

It is noteworthy that the juvenile black bear samples in this study were collected at only 0.25 years of age, which corresponds to a developmental/life-stage period potentially overlapping with weaning and milk-to-solid dietary transition. between juvenile and adult bears should be interpreted primarily as a life-stage comparison rather than chronological age alone [13]. Compared with adult individuals, in which the fecal microbiota has already reached maturity and stability, the fecal environment of juvenile individuals may be more susceptible to invasion and colonization by exogenous pathogenic bacteria, for example through feed, drinking water, air, or direct contact with keepers and the captive environment [22]. These life-stage-linked dietary transitions may also alter gut conditions and exposure routes (e.g., via feed and drinking water), which could jointly contribute to the observed differences in fecal MBPD-annotated potentially pathogenic bacterial communities. This inference is also corroborated by the ordination analysis results of this study: both NMDS and PCoA analyses showed that the fecal pathogenic bacterial community composition of captive juvenile black bears was relatively similar to that of captive brown bear samples from another zoo, with a certain degree of overlap in their distribution in the ordination space, whereas a clearer separation was observed from the captive adult black bear samples within the same zoo. Taken together, these findings suggest that what we observe as an “age effect” likely reflects life-stage-associated developmental transitions (including weaning and dietary shifts), rather than chronological age in isolation, and that age may contribute in conjunction with weaning/diet-associated factors in this process [23]. In other words, the fecal microbiota of juvenile black bears has not yet formed a stable species-specific structure, and its pathogenic bacterial composition pattern may be shaped more by external environmental exposure than strictly determined by inherent characteristics of the host species or the zoo in which it resides.

With respect to the exploratory neutral community model (NCM) analysis, the fitting results showed that platform-reported R^2^ values were below 0.5 in all three groups—captive brown bears, captive juvenile black bears, and captive adult black bears. These values describe the fit of the NCM to the observed occurrence–abundance relationships but do not, by themselves, identify deterministic or stochastic processes. Although the platform-reported Nm values differed numerically among groups, no formal statistical comparison was performed. Therefore, the NCM results are presented as exploratory model-fitting patterns and should not be interpreted as evidence of differences in stochastic processes, deterministic processes, dispersal, migration, or community assembly mechanisms among groups.

In summary, this study systematically analyzed the fecal pathogenic bacterial community structure and exploratory occurrence–abundance patterns of captive brown bears from Yangqi Mountain Zoo and captive adult and juvenile Asiatic black bears from Jinan Wildlife Zoo. The results indicate that zoonotic pathogenic bacteria, represented by *Escherichia coli*, occupied a relatively high proportion of abundance in the fecal MBPD-annotated potentially pathogenic bacterial communities of all three groups. Based on these findings, we suggest that while captive environments provide stable rearing conditions for bears, they may also, to some extent, increase the risk of colonization and enrichment of zoonotic pathogenic bacteria such as *E. coli* in the host feces, thereby potentially increasing the likelihood of gastrointestinal disease in captive individuals. In routine husbandry management, it is recommended to strengthen the cleaning and disinfection of rearing environments and contact items (e.g., through routine measures such as alcohol disinfection) to reduce the opportunities for transmission of zoonotic pathogenic bacteria to the feces of captive individuals. The findings of this study are expected to provide a useful reference for the scientific husbandry management and disease prevention and control of captive Asiatic black bears and brown bears.

This study has several limitations. Sequencing of the V3–V4 region provides limited taxonomic resolution and cannot reliably identify bacteria at the species or strain level or assess their functional potential. In addition, the total sample size was limited, particularly for the juvenile black bear group (*n* = 3), which may reduce the stability and generalizability of the observed patterns. In addition, sex information was unavailable for some individuals, preventing a full evaluation of sex-related effects. Potentially relevant factors, including sampling season, health status, medication exposure, enclosure/co-housing conditions, and diet, could not be fully evaluated because of the limited sample size and/or incomplete metadata. Future studies using higher-resolution sequencing and larger, balanced sample groups with detailed host and husbandry metadata are needed to validate these findings.

## 5. Conclusions

This study characterized fecal MBPD-annotated potentially pathogenic bacterial communities in captive brown bears and black bears (adult and juvenile) via 16S rRNA sequencing and the MPBD database. Zoonotic pathogens, especially *Escherichia coli*, dominated all three groups, with adult black bears showing the highest abundance and diversity, while juveniles resembled brown bears, reflecting their immature fecal microbiota. Neutral community model fitting showed low explanatory power (R^2^ < 0.5) across groups, indicating that non-neutral influences are likely involved, but the relative contribution of captive management cannot be concluded as the primary deterministic driver for fecal pathogenic bacterial community assembly. These findings provide baseline data on fecal pathogenic bacteria in captive ursids and warrant future studies incorporating wild comparators and longitudinal sampling to further clarify the roles of captivity, host age, and species in shaping these communities.

## Data Availability

The raw sequencing data have been submitted to the China National Center for Bioinformation (CNCB-NGDC) under BioProject accession numbers PRJCA070175 and PRJCA070170.

## Appendix A

**Table A1:** Specimen information for black bears and brown bears.

Species	Collection Site	Number	Gender	Age (Year)
Brown bear	Yangqishan Zoo, Chaohu City, Anhui Province, China	UA1	Female	5
Brown bear	Yangqishan Zoo, Chaohu City, Anhui Province, China	UA2	Female	5
Brown bear	Yangqishan Zoo, Chaohu City, Anhui Province, China	UA3	Female	5
Brown bear	Yangqishan Zoo, Chaohu City, Anhui Province, China	UA4	Female	5
Brown bear	Yangqishan Zoo, Chaohu City, Anhui Province, China	UA5	Female	5
Brown bear	Yangqishan Zoo, Chaohu City, Anhui Province, China	UA6	Female	5
Brown bear	Yangqishan Zoo, Chaohu City, Anhui Province, China	UA7	Male	5
Brown bear	Yangqishan Zoo, Chaohu City, Anhui Province, China	UA8	Male	5
Brown bear	Yangqishan Zoo, Chaohu City, Anhui Province, China	UA9	Male	5
Brown bear	Yangqishan Zoo, Chaohu City, Anhui Province, China	UA10	Male	5
Black bear	Jinan Wildlife Park, Jinan City, Shandong Province, China	UT1	Male	4.5
Black bear	Jinan Wildlife Park, Jinan City, Shandong Province, China	UT2	Male	4.5
Black bear	Jinan Wildlife Park, Jinan City, Shandong Province, China	UT3	Male	4.5
Black bear	Jinan Wildlife Park, Jinan City, Shandong Province, China	UT4	Male	4.5
Black bear	Jinan Wildlife Park, Jinan City, Shandong Province, China	UT5	Male	4.5
Black bear	Jinan Wildlife Park, Jinan City, Shandong Province, China	UT6	Male	4.5
Black bear	Jinan Wildlife Park, Jinan City, Shandong Province, China	UT7	Male	4.5
Black bear	Jinan Wildlife Park, Jinan City, Shandong Province, China	UT8	Unknow	0.25
Black bear	Jinan Wildlife Park, Jinan City, Shandong Province, China	UT9	Unknow	0.25
Black bear	Jinan Wildlife Park, Jinan City, Shandong Province, China	UT10	Unknow	0.25
